# Direct measurements of ferric reductase activity of human 101F6 and its enhancement upon reconstitution into phospholipid bilayer nanodisc

**DOI:** 10.1016/j.bbrep.2020.100730

**Published:** 2020-01-17

**Authors:** Mohammed El Behery, Mika Fujimura, Tetsunari Kimura, Motonari Tsubaki

**Affiliations:** Department of Chemistry, Graduate School of Science, Kobe University, Nada-ku, Kobe, Hyogo, 657-8501, Japan

**Keywords:** Human 101F6 protein, Cytochrome *b*_561_, Ferric reductase activity, Nanodisc reconstitution, FAC, Nitroso-PSAP

## Abstract

We studied human 101F6 protein to clarify its physiological function as a ferric reductase and its relationship to tumor suppression activity. We found for the first time that purified 101F6 both in detergent micelle state and in phospholipid bilayer nanodisc state has an authentic ferric reductase activity by single turnover kinetic analyses. The kinetic analysis on the ferrous heme oxidation of reduced 101F6 upon the addition of a ferric substrate, ferric ammonium citrate (FAC), showed concentration-dependent accelerations of its reaction with reasonable values of *K*_M_ and *V*_max_. We further verified the authenticity of the ferric reductase activity of 101F6 using nitroso-PSAP as a Fe^2+^-specific colorimetric chelator. 101F6 in nanodisc state showed higher efficiency for FAC than in detergent micelle state.

## Highlights

• Human tumor suppressor 101F6 protein was reconstituted into nanodisc.
• 101F6 functions as a ferric reductase both in detergent micelle and in nanodisc.
• 101F6 in nanodisc showed higher efficiency in the reductase activity.

## 1 Introduction

Human *101F6* gene in chromosome 3p21.3 was predicted to have a tumor suppression activity [1,2]. Indeed, the growth of lung cancer cells was inhibited upon the forced expression of the *101F6* gene *via* the induction of apoptosis and/or autophagy [2,3]. The *101F6* gene was found to code for a transmembrane protein (101F6 protein) that is a member of the cytochrome *b*_561_ protein family [1] and contains two hemes *b* [4]. The subcellular localization of 101F6 protein was found in *endoplasmic reticulum* membranes or in small vesicles including endosomes [4]. We and other groups have conducted detailed studies on the heterologously expressed 101F6 at the molecular level [[5], [6], [7]], but its physiological functions are still obscured.

Previously, detergents, e.g. n-octyl-β-d-glucopyranoside (β-OG) and n-dodecyl-β-D-maltoside (DDM) were used to study 101F6 in solubilized micelle state. The use of β-OG and DDM, however, can affect the stability of 101F6 and may interfere with its biochemical and biophysical measurements. To avoid these problems, we tried for the first time the reconstitution of 101F6 into phospholipid bilayer nanodisc. Nanodisc consists of phospholipid bilayer wrapped by two molecules of a membrane scaffold protein (MSP). Incorporation of membrane proteins into nanodisc offers great advantages than in liposomes and in detergent micelles by enabling the access on both sides of phospholipid bilayer membranes [8,9]. Chemical and biophysical resemblances of nanodiscs to cell membranes will keep them soluble in aqueous media and maintain the protein stability and functional activity [10].

Iron is important for various biological processes such as transport of dioxygen and electron transfer reactions [11]. Iron accumulation in different tissues would cause several diseases due to the generation of free radicals that damage cellular components [12,13]. Most of the iron in the environments at physiological pH exists as a ferric state (Fe^3+^) [14].

Various organisms utilize the action of ferric reductase enzymes that reduce Fe^3+^ to ferrous state (Fe^2+^) at enterocytes of intestines. Duodenal cytochrome *b* (Dcytb) was found to have such activity [14,15], Then, Fe^2+^ can be absorbed into the cells by divalent metal transporter 1 (DMT1) [11,14]. It was proposed later that most cytochromes *b*_561_ proteins, including Dcytb, may function as a ferric reductase to facilitate the iron uptake by the cells or its transfer within the cells [16]. These include chromaffin granules cytochromes *b*_561_ (CGcytb), lysosomal cytochrome *b*_561_ (Lcytb) [17], stromal cell-derived receptor 2 (SDR2) [11], and 101F6 [4]. Plant *Arabidopsis* TCytb/CYB561B1 [18] was also proposed to have the ferric reductase activity.

In the present study, we focused on the ferric reductase activity of purified 101F6 in detergent micelle state and in nanodisc state. We found for the first time that purified 101F6 has an authentic ferric reductase activity by directly observing the electron transfer from the ferrous heme to ferric-chelate substrates and that the incorporation of 101F6 into nanodisc enhanced the efficiency of the reaction for the ferric-chelate substrates.

## 2 Materials and methods

### 2.1 Materials

Plasmid (pET28a-MSP1D1ΔH5) was purchased from Addgene (USA), used for transformation of *E. coli* BL21(DE3) strain. 1,2-dimyristoyl-*sn*-glycero-3-phosphocholine (DMPC) was obtained from Tokyo Chemical Industry, Japan. Bio-beads SM-2 was obtained from BIORAD, USA. HiTrap^TM^ Desalting Column was purchased from GE Healthcare Japan Ltd. Ferric ammonium citrate (FAC) was obtained from Wako Pure Chemical Industries, Ltd, Japan. 2-Nitroso-5- [*N*-*n*-propyl-*N*-(3-sulfopropyl) amino] phenol (Nitroso-PSAP) and DDM were obtained from Dojin Chemical Research Laboratory, Japan.

### 2.2 Expression and purification of 101F6 and membrane scaffold protein

Expression and purification of 101F6 were conducted by employing alcohol-assimilating yeast *Pichia pastoris* cells and pPICZB-101F6-His_8_ plasmid [5,6]. In brief, the human *101F6* gene was incorporated into the *Pichia* genome and successful transformants were selected by zeocin-resistance screening. After the culture, the microsomal fraction was prepared from harvested *Pichia* cells. Then, the microsomal fraction was solubilized with DDM. Purification of 101F6 was conducted by using a Ni-NTA-Sepharose affinity column [5]. MSP1D1ΔH5 protein was expressed using the expression system with *E. coli* BL21 (DE3) strain and purified by a Ni-NTA Sepharose affinity column [8,19].

### 2.3 Reconstitution of 101F6 in nanodisc

MSP1D1ΔH5 was added to the phospholipid (DMPC) solution to yield the desired MSP:DMPC ratio of 2:160. The mixture solution was incubated at room temperature with gentle stirring for 30 min. Then, target 101F6 protein (in 50 mM potassium phosphate buffer, 0.1% DDM, pH 7.0) was added to give a specific MSP1D1ΔH5:DMPC:101F6 ratio. After 1 h incubation at room temperature, detergents were removed by an overnight (12–16 h) treatment with 100–200 mg of wet Bio-beads SM-2 with gentle stirring at room temperature. Bio-beads were then removed from the solution and the solution containing nanodisc mixture was filtered through a 0.22-μm filter.

### 2.4 Size exclusion chromatography (SEC**)**

The nanodisc mixtures were then injected onto an SEC column (Superdex^TM^ 200 10/300 GL column connected with an AKTA Pure chromatography system; GE Healthcare). The column was pre-equilibrated with 2 column volumes of Milli-Q water followed by 2 column volumes of equilibration buffer (20 mM Tris-HCl, 0.1 M NaCl, pH 7.4) with a flow rate of 0.25 mL/min. 101F6-MSP1D1ΔH5 nanodisc was eluted from the column with the equilibration buffer with a flow rate of 0.4 mL/min while monitoring A_280_ for total protein and A_416_ for 101F6. Fractions containing 101F6-MSP1D1ΔH5 nanodisc were collected for spectral measurements and SDS-PAGE analysis. In our study, ascorbate was used as a physiological electron donor for 101F6 to confirm its quality and structural intactness. However, ascorbate cannot reduce 101F6 completely. Therefore, sodium dithionite was used to prepare the fully-reduced form of 101F6.

### 2.5 Measurement of ferric reductase activity by ferrous heme oxidation of reduced 101F6

In the present study, we tried to measure the ferric reductase activity of 101F6 by observing the acceleration of the oxidation of the ferrous heme moiety upon the addition of a ferric substrate in single turnover kinetic experiments under anaerobic conditions. We first measured the autoxidation process of the ferrous heme of reduced 101F6 in detergent micelle state. For the analyses, it was important to remove dioxygen from the buffer. First, 50 mL of buffer (50 mM Tris-HCl, 10% glycerol, pH 7.0) was bubbled with pure nitrogen gas for 1 h. Then, solid DDM (final 0.1%) was added anaerobically to the buffer. Thus prepared deoxygenated buffer was used for all of the measurements. A fully reduced 101F6 was prepared by adding a small amount of sodium dithionite anaerobically and was, then, injected anaerobically onto a HiTrap^TM^ desalting column previously equilibrated with the deoxygenated buffer. The eluate from the column was transferred anaerobically into a deoxygenated quartz cell and its UV–visible absorption spectrum was measured (UV-2400PC, Shimadzu Corporation, Japan) continuously in a repeated scanning mode. The decay of the ferrous heme to the oxidized form (autoxidation) measured by absorbance at 427 nm was fitted by a single exponential function.

Then, we analyzed the ferrous heme oxidation upon the addition of ferric substrate to the reduced 101F6 prepared as described above. Immediately after its preparation, its UV–visible absorption spectrum was measured. Then, a fixed volume (5 μL) of deoxygenated FAC solution was added anaerobically. The time-dependent spectral changes were measured in a repeated scanning mode and the absorbance changes at 427 nm were fitted by a double exponential function. Measurements and analyses of ferric reductase activity of 101F6 in nanodisc state were conducted similarly but in the absence of DDM in the buffer.

### 2.6 Nitroso-PSAP assay

In this assay, nitroso-PSAP, a ferrous ion-specific colorimetric reagent, was used to detect the formation and the concentration of ferrous ion produced by the ferric reductase activity of 101F6. Fe^2+^-nitroso-PSAP complex has an absorption peak at 756 nm with its molar extinction coefficient ε_756_ = 45,000 M^−1^ cm^−1^ [20]. In the assay procedures, just after the preparation of the reduced form of 101F6 either in detergent micelle state or in nanodisc state, its UV–visible absorption spectrum was measured anaerobically. Then, immediately, ferric substrate FAC and nitroso-PSAP were added, and the spectral changes were measured in a repeated scanning mode. The decay in absorbance at 561 nm, the β-band peak of reduced 101F6, and the increase in absorbance at 756 nm due to the formation of Fe^2+^-nitroso-PSAP were plotted against time and the plots were analyzed by a single exponential function.

## 3 Results

### 3.1 Measurement of ferric reductase activity in detergent micelle state

To measure the ferric reductase activity of purified 101F6 by single turnover experiments, dioxygen should be removed from the reaction medium or, at least, being kept at the lowest level. The presence of excess dioxygen in the measuring buffer would cause a significant acceleration of the autoxidation of ferrous heme of 101F6 during the activity measurements. The autoxidation measurement in our anaerobic condition showed that the ferrous heme became oxidized very slowly following a single exponential decay (*k* = 0.082 ± 0.002 min^−1^) (Fig. S1) and the results were reproducible. Thus, we concluded that the reduced 101F6 in detergent micelle state was stable enough to measure its enzymatic activity. Then, we examined the effects of the addition of FAC anaerobically to the reduced 101F6 in detergent micelle state. The addition of FAC accelerated the oxidation of the ferrous heme moiety significantly and the acceleration was concentration-dependent; *i.e.*, the rate of ferrous heme oxidation increased as the FAC concentration increased (Fig. S2A). By analyzing the decay process by a double exponential function, in which the rate constant (*k*_2_) value for its slower decay was fixed to that obtained for the ferrous heme autoxidation, we regarded the apparent rate constant *k*_1_ of the ferrous heme oxidation as the ferric reductase activity. Then the apparent rate constant *k*_1_ values were plotted against the FAC concentrations, showing that the plot followed a hyperbolic saturation. By fitting with the Michaelis-Menten-type equation (*V* = *V*_max_ × S/(*K*_M_ + S)), we could obtain *V*_max_ = 0.73 ± 0.14 min^−1^ and *K*_M_ = 23 ± 13.5 μM for purified 101F6 in detergent micelle state (Fig. S2B).

### 3.2 Reconstitution of 101F6-MSP1D1ΔH5 nanodisc

**Fig. 1:**
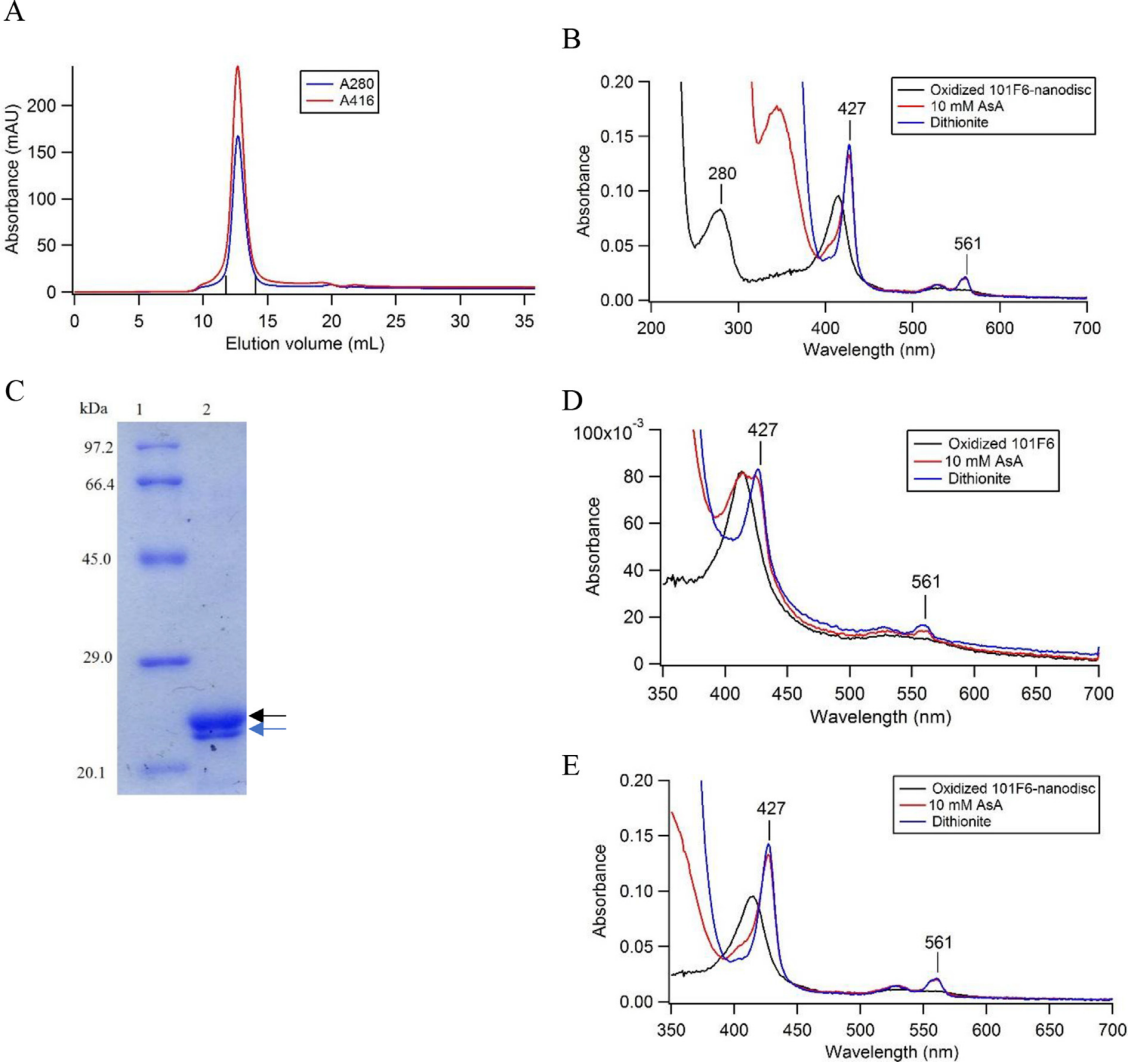
Preparation and analyses of 101F6-MSP1D1ΔH5 nanodisc. (A) Elution profile from the size exclusion chromatography for the reconstituted 101F6-MSP1D1ΔH5 nanodisc prepared at the mixing ratio of 2:160:1. Fractions between the two vertical lines were collected with a peak *K*_av_ = 0.36. *K*_av_ value was calculated by the equation *K*_av_ = (*V*_e_-*V*_o_)/(*V*_c_-*V*_o_), in which *V*_e_ as elution volume of substance, *V*_o_ as void volume, and *V*_c_ as total packed column volume. (B) UV– visible absorption spectra of the purified 101F6-MSP1D1ΔH5 nanodisc after the size exclusion chromatography. (C) SDS-PAGE analysis of the purified 101F6-MSP1D1ΔH5 nanodisc. Lane (1), LMW marker; lane (2), 101F6-MSP1D1ΔH5 nanodisc (MSP1D1ΔH5, 22.1 kDa, black arrow; 101F6, 26.0 kDa, blue arrow; their apparent positions being reversed due to the very hydrophobic nature of 101F6). (D, E) Comparison of visible spectra of 101F6 in detergent micelle state (D) and in nanodisc state (E) after standing at room temperature for 3 days. Red line, reduced by 10 mM ascorbate; blue line, reduced by sodium dithionite.

Since 101F6 is a very hydrophobic membrane-spanning protein and, therefore, to clarify its physiological function as a ferric reductase and its possible relationship to tumor suppression activity, it is very necessary to study the ferric reductase activity in native environments instead of detergent micelle state. Accordingly, we decided to reconstitute purified 101F6 into nanodisc consisting of DMPC and MSP1D1ΔH5. We searched for optimized conditions for the reconstitution of 101F6-MSP1D1ΔH5 nanodisc by changing the concentration of DMPC and MSP1D1ΔH5, while the concentration of 101F6 being kept constant. Some unsuccessful results with a higher or a lower MSP:DMPC ratio were shown in Fig. S3, where no specific peak for the 101F6-containing nanodisc appeared in the chromatograms of the size exclusion chromatography. We found the ratio of MSP1D1ΔH5:DMPC as 2:160 to be the most optimized one. Then, we searched for optimized concentrations of 101F6 with the MSP1D1ΔH5:DMPC ratio being fixed at 2:160. Finally, we found the ratio of MSP1D1ΔH5:DMPC:101F6 as 2:160:1 to be the best. Lowering of 101F6 concentration caused a slight increase of aggregates as shown in Fig. S4. Fractions containing 101F6-MSP1D1ΔH5 nanodisc were eluted as a single peak from the size exclusion chromatography with a retention volume and an average distribution coefficient (*K*_av_) as 13.12 ± 0.3 mL and 0.36 ± 0.02, respectively (Fig. 1A). By measuring the UV–visible absorption spectrum for purified 101F6-MSP1D1ΔH5 nanodisc (Fig. 1B), we found followings. (1) A relative increase in absorbance at 280 nm compared to that of 101F6 in detergent micelle was due to the presence of MSP1D1ΔH5 in the nanodisc. (2) Reducibility of the 101F6-MSP1D1ΔH5 nanodisc by ascorbate was well-conserved and even higher than in detergent micelle state indicating that 101F6 incorporated into nanodisc had a native structure and could accept electrons from ascorbate efficiently. (3) The percentage of the fully reconstituted nanodisc and the yield of the reconstitution for 101F6 were estimated as 76.6 ± 6.3% (corresponding to the molar ratio of 101F6:MSP = 1:2.6 ± 0.22) and 25.3 ± 1.5%, respectively.

From the SDS-PAGE analysis (Fig. 1C), we found two protein bands for MSP1D1ΔH5 and 101F6 at the expected positions inferred from those of purified samples, respectively. The estimated protein band intensities with ImageJ analysis showed satisfactory results (101F6:MSP1D1ΔH5 ratio as 1:3.1). We assumed that 101F6 can be incorporated as a monomer or a homodimer into the nanodisc based on the previous reports on the diameter of MSP1D1ΔH5-containing empty nanodisc [19] and the fact that Dcyt*b* [21] and cytochrome *b*_561_ from *Arabidopsis thaliana* [22] have a tendency to form a homodimer. Such a homodimer can be theoretically incorporated into a single MSP1D1ΔH5-nanodisc. In addition, to evaluate the structure of the 101F6-MSP1D1ΔH5 nanodisc, measurements by atomic force microscopy (AFM) were performed under aqueous buffer conditions (20 mM Tris-HCl at pH 8.0, 0.1 M NaCl). As shown in Fig. S5, a homogenous population of structures with 5–10 nm in height or diameter was observed, being consistent with the expected size for the 101F6-MSP1D1ΔH5 nanodisc.

### 3.3 Stabilization of 101F6 in nanodisc environments

We found that the stability of 101F6 in a nanodisc state was much better than in detergent micelle state. Heme-reducibility of 101F6 by ascorbate or by dithionite was severely damaged in detergent micelle state during longer storage (3 days) at room temperature (Fig. 1D). It might be also noted that there was a significant increase in absorbance in a shorter wavelength region, indicating the increase in turbidity due to the protein denaturation. On the other hand, 101F6 in nanodisc state showed high heme-reducibility by ascorbate and no denaturation occurred even after longer storage (3 days) at room temperature (Fig. 1E).

### 3.4 Measurements of ferric reductase activity in nanodisc state

**Fig. 2:**
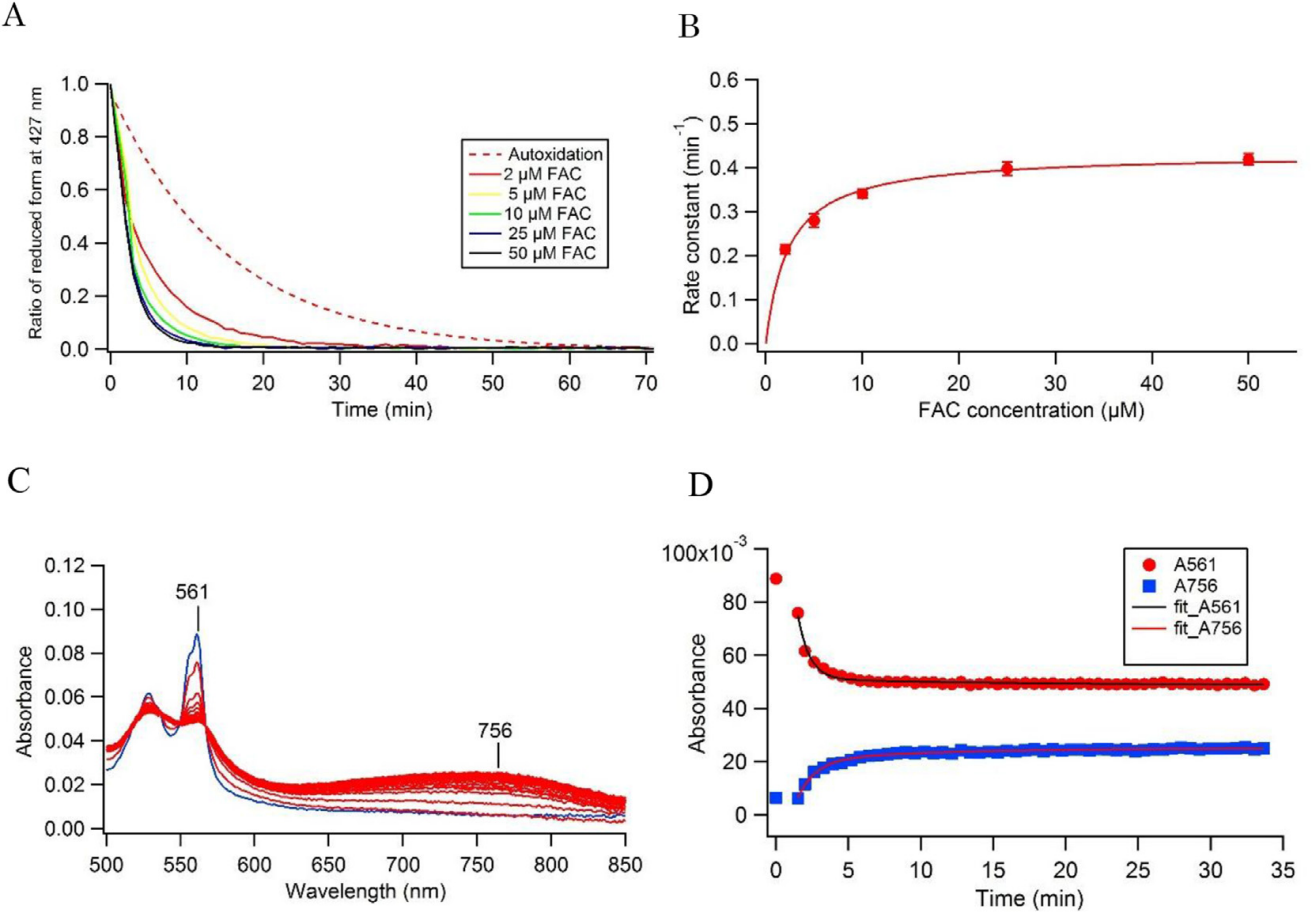
Measurements of ferric reductase activity of 101F6-MSP1D1ΔH5 nanodisc. (A) Oxidation of the ferrous heme of reduced 101F6-MSP1D1ΔH5 nanodisc measured by absorbance changes at 427 nm upon addition of various concentrations of FAC anaerobically. Autoxidation process of the ferrous heme in 101F6-MSP1D1ΔH5 nanodisc (broken line) was shown for a visual purpose. The decay was fitted by a double exponential function; y = y_0_ + [*A*_1_ × exp{-*k*_1_(t-t_0_)} + *A*_2_ × exp{–*k*_2_(t-t_0_)}]. (B) A plot of the reaction rate constants *k*_1_ (calculated from the data shown in panel A) against FAC concentrations and its analysis by the Michaelis-Menten equation. (C) Measurements of spectral change in a repeated scanning mode for 101F6-MSP1D1ΔH5 nanodisc (2 μM) upon addition of 40 μM nitroso-PSAP and 10 μM FAC. Blue line, the reduced form of 101F6-nanodisc just after the elution from the deoxygenated HiTrap^TM^ desalting column under anaerobic conditions; red lines, progress of the formation of Fe^2+^-nitroso-PSAP complex and the heme oxidation after addition of 40 μM nitroso-PSAP and 10 μM FAC. (D) Absorbance changes at 561 nm for reduced 101F6 and at 756 nm for Fe^2+^-nitroso-PSAP complex were plotted against time and fitted by a single exponential function: y = y_0_ + *A* × exp{-*k*_1_(t-t_0_)}.

We measured the autoxidation process of the ferrous heme moiety for the reduced form of 101F6 in nanodisc state in a similar way (but in the absence of detergent DDM). We found that, in nanodisc state, the apparent rate constant for the heme oxidation (*k* = 0.066 ± 0.002 min^−1^) was lower than that in detergent micelle state (*k* = 0.082 ± 0.002 min^−1^) (Fig. S1), indicating the further stabilization of the ferrous heme moiety in nanodisc state. Then, the ferric reductase activity of 101F6 in nanodisc state was analyzed in a similar way (Fig. 2A). We found that kinetic values for *K*_M_ and *V*_max_ (*V*_max_ = 0.432 ± 0.01 min^−1^, *K*_M_ = 2.34 ± 0.34 μM) obtained for 101F6 in nanodisc state (Fig. 2B) were lower than those of 101F6 in detergent micelle state (*V*_max_ = 0.73 ± 0.14 min^−1^, *K*_M_ = 23 ± 13.5 μM). These results indicated that 101F6 in nanodisc state had much higher efficiency for FAC (*V*_max_/*K*_M_ = 0.185 min^−1^/μM) than 101F6 in detergent micelle state (*V*_max_/*K*_M_ = 0.032 min^−1^/μM). Based on these results, we concluded that the structure of the active site of 101F6 in detergent micelle state may be slightly changed from its native one in the cell membranes due to the complete substitution of the phospholipids moiety characterize by the ordered charged head groups and hydrophobic tails with less ordered detergent molecules having a shorter hydrophobic tail and a sugar head group. Therefore, the Fe^3+^ substrate FAC can bind to the active site of 101F6 in nanodisc in a higher affinity than in detergent micelle state. Thus, nanodiscs can provide native-like membrane environments suitable for maintaining the protein stability for a longer time even at room temperature and enabling the fine-tuning of the enzymatic specificity of 101F6.

### 3.5 Measurements of ferric reductase activity by nitroso-PSAP assay

Then, we analyzed the authenticity of the ferric reductase activity of 101F6 by using nitroso-PSAP. Upon addition of nitroso-PSAP (40 μM) and FAC (10 μM) anaerobically, the absorbance at 561 nm of the reduced 101F6 in nanodisc state decreased in its intensity while a broad band centered at 756 nm gained its intensity (Fig. 2C). However, the addition of nitroso-PSAP alone did not cause any oxidation of the ferrous heme (data not shown). These results indicated that the reduced 101F6 in nanodisc transfers its electrons to FAC to reduce ferric ion to ferrous state, leading to the formation of Fe^2+^-nitroso-PSAP complex. It should be noted that the rate of the electron transfer (*k* = 1.25 ± 0.04 min^−1^) from the ferrous heme to ferric ion seemed faster than the formation of Fe^2+^-nitroso-PSAP complex (*k* = 0.46 ± 0.02 min^−1^) (Fig. 2D). We reasoned this observation as follows; upon reduction of FAC at the substrate-binding site of 101F6, the produced Fe^2+^-ammonium citrate was stable enough at the substrate-binding site and/or the release of Fe^2+^ from the ammonium citrate moiety was very slow. Therefore, the formation of Fe^2+^-nitroso-PSAP complex might be rate-limited by the exchange of chelate ligands.

## 4 Discussion

It was proposed previously that the cytochrome *b*_561_ family members have the ferric reductase activity more or less [17,18,21]. One of these studies involved an expressed cytochrome *b*_561_ homologous in yeast strains that lack the reductase enzymes [17,21]. Another study involved the investigation of tonoplast Cytb561 (TCytb) being expressed in microsomal membranes of yeast cells [18]. In these studies, ferrozine was used as a Fe^2+^ chelator to form a colored complex with an absorbance peak at 562 nm [23]. Since cytochrome *b*_561_ has a peak centered at 561 nm in the reduced form, its direct observation may be interfered with the peak of Fe^2+^-ferrozine complex. Further, involvements of other enzymes having a ferric reductase activity were not completely ruled out, since these measurements were conducted using intact cells or microsomal membranes. Therefore, in all these previous studies, the authenticity of ferric reductase activity of most cytochrome *b*_561_ seemed not fully established. Further, without the measurements of cytochromes *b*_561_ contents in these preparations (in intact cells and/or in microsomal membranes), it is very difficult to evaluate the enzymatic efficiency or specificity of cytochrome *b*_561_ participating in the ferric reductase activity.

We have succeeded to show at the molecular level that our highly purified 101F6 has an authentic ferric reductase activity using FAC as a ferric-substrate both in detergent micelle state and in nanodisc state. Since 101F6 is a member of cytochrome *b*_561_ family, our success would be of great importance for the understanding of the physiological roles of 101F6 in iron metabolism in various organisms including human beings. Based on our present findings that 101F6 functions as a ferric reductase, we can speculate that this physiological function has some roles for its unexplained function as a tumor suppressor protein, in which 101F6 somehow suppresses and inhibits the growth of human cancer cells. We and others have proposed that 101F6 is localized in endosome membranes to transfer electrons from cytosolic ascorbate (as a physiological electron donor) to the intravesicular side [4], (Asada *et a*l., unpublished results). Thus, it is very likely that the physiological role of 101F6 might be the reduction of ferric-substrate to ferrous state in endosomes to facilitate the transportation of iron to the cytoplasm through DMT1.

Previous studies revealed that a new type of cell death called ferroptosis [24] exists, which depends on the presence and metabolism of intracellular iron. Ferroptosis is induced by the generation of toxic reactive oxygen species (ROS) in the presence of ferrous ion followed by the accumulation of lethal lipid peroxidation products [24,25]. Based on the ferric reductase activity of 101F6, a following possible scenario can be made. Forced expression of 101F6 in human cancer cells in the presence of enough amounts of ascorbate results in the accumulation of ferrous ion in the cytoplasm. The reaction of Fe^2+^ with hydrogen peroxide, H_2_O_2_, would result in the formation of lethal ROS by Fenton reaction [26], which would induce cancer cell death *via* ferroptosis. It is well known that the production of H_2_O_2_ is higher in cancer cells compared to normal cells [27] but cancer cells have a lower ability to eliminate H_2_O_2_ due to the low level of expression in catalase and glutathione peroxidase [28].

It is known that in the human endosomes another form of ferric reductase, Steap3, exists. Steap3 is known as a transmembrane protein containing a single heme *b* and an FAD by receiving electrons from cytosolic NADPH. Importantly, Steap3 is known to be very critical to the metal homeostasis and linked to multiple diseases including cancer [29]. We need further clarification of the physiological function of 101F6 and Steap3 as ferric reductase enzymes. There may be a direct connection between 101F6 and Steap3 by forming a heterodimer in the endosomal membranes, although the source of electrons is different from each other. We are currently pursuing a possibility that 101F6 can directly or indirectly donate electrons to Steap3, or *vice versa*, to function as ferric reductases in endosomes.

## Funding acknowledgments

This work was supported by Grant-in-Aid for Scientific Research (C) (25440048 and 16K07323 to MT) from Japan Society for the Promotion of Science.

## CRediT authorship contribution statement

**Mohammed El Behery:** Investigation, Methodology, Formal analysis, Data curation, Writing - original draft. **Mika Fujimura:** Investigation, Methodology, Formal analysis, Data curation. **Tetsunari Kimura:** Supervision, Methodology. **Motonari Tsubaki:** Conceptualization, Supervision, Writing - review & editing.

## Appendix A Supplementary data

Data Profile Data Profile
